# An open secret in porcine acute myocardial infarction models: The relevance of anaesthetic regime and breed in ischaemic outcomes

**DOI:** 10.3389/fvets.2022.919454

**Published:** 2022-10-24

**Authors:** Núria Solanes, Joaquim Bobi, Marta Arrieta, Francisco Rafael Jimenez, Carmen Palacios, Juan José Rodríguez, Mercè Roqué, Carlos Galán-Arriola, Borja Ibañez, Xavier Freixa, Ana García-Álvarez, Manel Sabaté, Montserrat Rigol

**Affiliations:** ^1^Hospital Clínic, Institut d'Investigacions Biomédiques August Pi i Sunyer (IDIBAPS) and Cardiology Department, Institut Clínic Cardiovascular, Universitat de Barcelona, Barcelona, Spain; ^2^Experimental Cardiology Department, Erasmus MC University Medical Centre, Rotterdam, Netherlands; ^3^Centro Nacional de Investigaciones Cardiovasculares Carlos III, CIBER de enfermedades cardiovasculares (CIBERCV), IIS- Fundación Jiménez Díaz Hospital, Madrid, Spain; ^4^Bioresearch and Veterinary Services, The University of Edinburgh, Edinburgh, United Kingdom

**Keywords:** myocardial infarction, anaesthesia, pig breed, porcine models, translational research

## Abstract

Large animal models of acute myocardial infarction (AMI) play a crucial role in translating novel therapeutic approaches to patients as denoted by their use in the right-before-human testing platform. At present, the porcine model of AMI is used most frequently as it mimics the human condition and its anatomopathological features accurately. We want to describe to, and share with, the translational research community our experience of how different anaesthetic protocols (sevoflurane, midazolam, ketamine+xylazine+midazolam, and propofol) and pig breeds [Large White and Landrace x Large White (LLW)] can dramatically modify the outcomes of a well-established porcine model of closed-chest AMI. Our group has extensive experience with the porcine model of reperfused AMI and, over time, we reduced the time of ischaemia used to induce the disease from 90 to 50 min to increase the salvageable myocardium for cardioprotection studies. For logistical reasons, we changed both the anaesthetic protocol and the pig breed used, but these resulted in a dramatic reduction in the size of the myocardial infarct, to almost zero in some cases (sevoflurane, 50-min ischaemia, LLW, 2.4 ± 3.9% infarct size), and the cardiac function was preserved. Therefore, we had to re-validate the model by returning to 90 min of ischaemia. Here, we report the differences in infarct size and cardiac function, measured by different modalities, for each combination of anaesthetic protocol and pig breed we have used. Furthermore, we discuss these combinations and the limited literature pertaining to how these two factors influence cardiac function and infarct size in the porcine model of AMI.

## Introduction

Acute myocardial infarction (AMI) and subsequent heart failure have been a leading cause of mortality and morbidity worldwide in the last decades (1). To address this, effective translation of novel therapeutic options to patients is crucial and multiple different expert recommendations have emphasized the key role of large-animal models of AMI as a right-before-patient test platform (2).

Presently, the swine (*Sus scrofa domestica*) is one of, if not, the most used large-animal model of AMI, primarily due to the significant anatomical similarities between pig and human hearts including their coronary vascular systems, for example minimal to absent collateral circulation (3). Due to these similarities, myocardial ischaemia/reperfusion in porcine models reproduces the pathophysiologic mechanisms of human acute ST-elevation myocardial infarction so accurately (3, 4) that the porcine model of AMI is used in research centers worldwide.

The basic concept behind the AMI model is to induce the lesion by occluding a coronary artery followed (or not) by reperfusion. Despite this basic concept, there are variations in the methodologies employed by different research groups/centers due to the specific purpose of the model and the expertise and limitations of each research group/center. Consequently, this has led to a number of porcine AMI models, each with slight variations and all at the expense of a standardized methodology, which hampers comparison between data from different groups and limits data pooling. Unfortunately, some of these variations in methodology have an influence on the outcome of ischaemia, resulting in a variable range of areas of necrosis (minimal to large myocardial infarctions). One influential factor is the anaesthetic protocol (5, 6), as some anaesthetics have cardioprotective properties (7). For example, the use of sevoflurane in human patients undergoing coronary artery bypass surgery has been linked to a reduction in the severity of the ischaemic insult. In pigs, some studies have also described cardioprotective effects associated with inhaled anaesthesia (8, 9) but it is not clear how consistent this protection is depending on the ischaemia duration induced by different methodologies. Less is known about the influence of different pig breeds, or familial lineages within a breed, on the susceptibility of the myocardium to ischaemia. Overall, the influence of the anaesthetic protocol and other variables, such as pig breed or age, on the extent and severity of the resultant ischaemia induced in porcine models of AMI is poorly described in the literature. This variation between methodologies is a topic of active discussion among translational scientists, despite a limited general interest in reporting failed pilot studies or negative results.

Here, we describe and share with the translational research community, our group's experience of how different porcine anaesthetic protocols and breeds can dramatically modify the severity of the resultant infarct in a well-established swine model of closed-chest AMI.

## Materials and methods

### Study groups

We compared pilot data (study A) with historical data generated by our group [studies B (10), C (11), and D (12) see below]. This study has been approved prior to its initiation, according to the European (2010/63/EU) and Spanish (RD 53/2013) regulations, by the Ethics Committee on Animal Experimentation of the Center for Comparative Medicine and Bioimage (CMCiB) and by *Generalitat de Catalunya* (reference 10802). All protocols used in this study comply with the principles of the 3Rs to prioritize animal welfare. All animal experimentation from study A was developed at the Centre for Comparative Medicine and Bioimage (CMCiB) of the Germans Trias i Pujol Research Institute (IGTP) (reference B9900005), Barcelona, Spain.

Study A was comprised of twenty Landrace x Large White female pigs (33.1 ± 2 kg; 3–4 months old) that underwent a reperfused AMI after different occlusion durations of the left anterior descending (LAD) coronary artery and under different anaesthetic regimens. Sub-groups were defined as the following: sub-group SEVO-50, anaesthesia maintained by inhalation with sevoflurane (0.6–3%) and 50 min of LAD coronary artery occlusion (*n* = 10); sub-group MIDA-50, anaesthesia maintained with intravenous (IV) midazolam (2–2.5 mg/kg/hour) and 50 min of LAD coronary artery occlusion (*n* = 1); sub-group MIDA-90, anaesthesia maintained with IV midazolam (2–2.5 mg/kg/hour) and 90 min of LAD coronary artery occlusion (*n* = 9) (Table 1, Study A).

**Table 1:** Pig breed, ischaemia time, anaesthetic regimens, and echocardiography or CMR results from the present study and previous publications of our research team.

	**Study A – 2020/2021**			**Historical data**		
	**SEVO – 50**	**MIDA – 50**	**MIDA – 90**	**Study B – 2017** **(9)**	**Study C – 2014 (10)**	**Study D – 2010 (11)**
Pig breed	Landrace x Large White	Landrace x Large White	Landrace x Large White	Large White	Landrace x Large White	Landrace x Large White
Occlusion duration (min)	50	50	90	50	90	90
Pre-medication	KXM (6; 4; 0.16 mg/Kg, respectively)	KXM (6; 4; 0.16 mg/Kg, respectively)	KXM (6; 4; 0.16 mg/Kg, respectively)	KXM (20; 2; 0.5 mg/Kg, respectively)	Azaperone (2 mg/kg)	Azaperone (2 mg/kg)
Anaesthesia (induction)	Propofol (1–2 mg/kg)	Propofol (1–2 mg/kg)	Propofol (1–2 mg/kg)	_________________	Sodium thiopental (30 mg/kg)	Sodium thiopental (30 mg/kg)
Anaesthesia (maintenance)	Sevoflurane (0.6–3 %) + Fentanyl (0.005–0.01 mg/kg/h)	Midazolam (2–2.5 mg/Kg/h) + Fentanyl (0.005–0.01 mg/kg/h)	Midazolam (2–2.5 mg/Kg/h) + Fentanyl (0.005–0.01 mg/kg/h)	KXM (2; 0.2; 0.2 mg/kg/h; respectively) + Fentanyl (0.007 mg/kg/h)	Propofol (10 mg/kg/min) + Fentanyl (0.025 mg/kg/h)	Propofol (10 mg/kg/min) + Fentanyl (0.025 mg/kg/h)
Animals* (sample size)	*n* = 9	*n* = 1	*n* = 9	*n* = 7	G15 min *n* = 6 G7d *n* = 6	Gic *n* = 4 Gte *n* = 4
LV function (method)	CMR	CMR	CMR	CMR	Intracardiac echocardiogram	Intracardiac echocardiogram
LV function (time points, days post-AMI)	90, 150	15	60, 120	7, 60	7, 21	7, 21
LVEDV (ml)	T90, 102.9 ± 15.5 T150, 129.7 ± 15.9	T15, 106.8	T60, 125 ± 14.1 T120, 156.2 ± 26.8	T7, 133.2 ± 17.0 T60, 201.3 ± 37.3	G15 min-T7, N/A G7d-T7, 30.4 ± 20.4 G15 min-T21, 46.3 ± 5 G7d-T21, 30.8 ± 22.7	G1-T7, 29.3 ± 0.6 G2-T7, 32.4 ± 4 G1-T21, 27.3 ± 3.9 G2-T21, 34.1 ± 5.9
LVESV (ml)	T90, 46.4 ± 9.3 T150, 63.2 ± 10	T15, 56.3	T60, 76.6 ± 14.4 T120, 96.1 ± 18.1	T7, 80.3 ± 16.9 T60, 126.5 ± 35.8	G15 min-T7, N/A G7d-T7, 14.3 ± 9.3 G15 min-T21, 22.3 ± 3.9 G7d-T21, 15.5 ± 11.8	G1-T7, 16.0 ± 0.2 G2-T7, 16.0 ± 0.2 G1-T21, 13.8 ± 1.3 G2-T21, 16.6 ± 3.2
LVEF (%)	T90, 54.7 ± 6.6 T150, 51.4 ± 3.2	T15, 47.2	T60, 39.1 ± 5.3 T120, 38.5 ± 3.4	T7, 40.2 ± 5.2 T60, 37.9 ± 6.3	G15 min-T7, N/A G7d-T7, 52 ± 3 G15 min-T21, 51 ± 9 G7d-T21, 52 ± 7	G1-T7, 45 ± 1 G2-T7, 50 ± 5 G1-T21, 49 ± 2 G2-T21, 51 ± 8
Infarct size (method)	CMR	CMR	CMR	CMR	Morphometry	Morphometry
Infarct size (% LV)	T90, 2.4 ± 3.9 T150, 1.8 ± 3.4	T15, 0	T60, 16.4 ± 4.6 T120, 15.7 ± 3.2	T7, 30.8 ± 5.5 T60, 19.1 ± 4.3	G15 min-T7, N/A G7d-T7, N/A G15 min-T21, 24 ± 4 G7d-T21, 21 ± 3	G1-T7, N/A G2-T7, N/A G1-T21, 19 ± 10 G2-T21, 22 ± 12

Values are represented as mean ± SD. SEVO, sevoflurane; MIDA, midazolam; AMI, acute myocardial infarction; CMR, cardiac magnetic resonance imaging; LV, left ventricle; LVEDV, left ventricular end-diastolic volume; LVEF, left ventricular ejection fraction; LVESV, left ventricular end-systolic volume; KXM, ketamine + xylazine + midazolam; N/A, non-applicable. G15min, group of control animals with vehicle administration 15 min after AMI induction; G7d, group of control animals with vehicle administration 7 days after AMI induction; Gic, group of control animals with intracoronary vehicle administration after AMI induction; Gte, group of control animals with transendocardial vehicle administration after AMI induction; ^*^ animals that survived AMI induction.

### Study A

The animals were acclimated for a minimum of 7 days and a maximum of 15 days in the research facilities prior to any procedures. A light–dark cycle of 12 h (with natural light through the glass, which guaranteed 12 h of light supplemented by artificial light if necessary), an ambient temperature of between 16 and 24°C, relative humidity between 30 and 70%, 15–20 air renewals per hour, and twice-daily feeding cycles along with *ad libitum* drinking water were provided for all animals.

The temperature was monitored using a rectal probe throughout the experimental procedure of AMI induction until the animal recovered from anaesthesia.

### Reperfused AMI procedure

Pre-medication of all the animals was comprised of intramuscular (IM) injection of ketamine (6 mg/kg), xylazine (4 mg/kg), and midazolam (0.16 mg/kg), and propofol (1–2 mg/kg) was administered intravenously for induction of anaesthesia. Anaesthesia was maintained with either sevoflurane (between 1.5 and 2.5 % but occasionally, to maintain stable hemodynamics with the required plane of anaesthesia, it was reduced to 0.6 % or raised to 3 %) or midazolam (2.0–2.5 mg/kg/h) depending on the study group (see above). An infusion of IV fentanyl (0.005–0.01 mg/kg/hour) during the procedure and buprenorphine (0.01 mg/kg, IM) toward the end of anaesthesia, to provide analgesia, were administered. Animals were orotracheally intubated, and mechanical ventilation was maintained during induction of AMI.

Following intubation, animals were mechanically ventilated (WATO EX-35). Ventilatory parameters were initially set as follows: volume-control, tidal volume 10 ml/kg, inspiratory fraction of oxygen 0.45, positive end-expiratory pressure 3 cm H_2_O, and respiratory rate adjusted to maintain PaCO_2_ within the physiologic range (35–45 mmHg).

Saline was administered intravenously at 10 ml/kg/hour and adjusted to maintain arterial pressure within normal values.

During the AMI induction procedure, attempts were made to maintain heart rate and mean arterial pressure between the ranges of 60–100 bpm and 60–70 mmHg, respectively, whilst maintaining an adequate plane of anaesthesia. However, it should be noted that blood pressure was monitored using a pressure cuff, the values of which are considered to be 10–15 mmHg lower compared to those obtained by using sensors placed in more invasive locations.

Briefly, the procedure consisted of percutaneous catheterization, *via* the femoral artery, of the LAD coronary artery, in which a balloon catheter was advanced, under fluoroscopic guidance, and inflated after the first diagonal branch, for 50 min or 90 min, followed by reperfusion for 15 min under anaesthesia prior to recovery. During coronary catheterization, appropriate anticoagulation was provided (an initial IV bolus of 3,000 IU heparin followed by boluses of 1,000 IU every 30 min). The day before induction of AMI, all animals received a loading dose of clopidogrel (150 mg/animal, PO) as an antithrombotic and this was continued for 2 days after the induction of AMI (clopidogrel 75 mg/animal/day, PO). During ischaemia, a continuous infusion of lidocaine (50 μg/kg/min, IV; sub-groups SEVO-50 and MIDA-50) or amiodarone (5.5 mg/kg/h, IV; sub-group MIDA-90) was administered to prevent malignant ventricular arrhythmias.

During follow-up, animals were monitored daily by a technician and weekly by a named veterinary surgeon. Animals were weighed weekly, and weight lost was monitored closely.

### Infarct outcomes by cardiac imaging and histology

Infarct size and left ventricle function were studied by cardiac magnetic resonance (CMR) imaging at the following time points: sub-group SEVO-50 at 90 and 150 days post-procedure, sub-group MIDA-50 at 15 days post-procedure, and sub-group MIDA-90 at 60 and 120 days post-procedure. CMR imaging was performed with a Vantage Galan 3.0 Tesla magnet (Canon Medical Systems Corporation, Tokyo, Japan), and each study consisted of a cine steady-state free-precision sequence to assess biventricular volumes and ejection fraction, and a late gadolinium-enhanced (LGE) sequence to determine infarct size. Images were analyzed by two independent, blinded investigators and processed with analysis software (QMass. Medis, Leiden, The Netherlands) as described previously (10).

After the last imaging time point animals were euthanized with an overdose of IV pentobarbital and hearts were collected for further detailed analyses. Briefly, the hearts and transverse slices were photographed to record gross lesions. Representative samples of the anterior wall of the left ventricle were collected from healthy and fibrotic areas (macroscopically pale areas), placed into fixative (10% buffered formalin), and processed by routine histological methods for the examination of haematoxylin and eosin (H&E)-stained tissue sections.

### Study groups B, C, and D

The results from study A were compared with control animals (with AMI and vehicle administration) from our previous research, studies B (10), C (11), and D (12). All animals were the same age, sex, and weight as those in study A at the beginning of the study. The procedure used to induce a reperfused AMI was the same in all the studies but used different times of duration of occlusion and different anaesthetic protocols depending on the aim of the study and the research center where the study was performed (Table 1). In summary, in study B we occluded the LAD coronary artery for 50 min and the anaesthetic protocol consisted of a combination of ketamine, xylazine, and midazolam (2.0, 0.2, and 0.2 mg/kg/h, respectively, IV) to induce and maintain general anaesthesia, and in studies C and D the LAD coronary artery was occluded for 90 min and anaesthesia was induced with sodium thiopental (30 mg/kg) and maintained with propofol (10 mg/kg/min, IV). In all studies, analgesia was provided by continuous infusion of fentanyl (0.005–0.025 mg/kg/h, IV) during the procedure.

## Results

### AMI procedure mortality

Studies A, B, C, and D had 5, 12.5, 8, and 32% AMI-related mortality, respectively, due to irreversible ventricular fibrillation during AMI induction. The rest of the animals completed the whole study as planned.

### Study A. Temperature and haemodynamic data

Temperatures remained within the range of 36.5 ± 0.4°C in the SEVO-50 sub-group, 37.5 ± 0.1°C in the MIDA-50 sub-group, and 36.6 ± 0.3°C in the MIDA-90 sub-group. These temperature ranges were considered acceptable for anaesthetized pigs.

The mean heart rate during ischaemia time (minutes of anterior descending coronary occlusion) in the SEVO-50 sub-group was 74.5 ± 9.3bpm, in the MIDA-50 sub-group it was 91.5 ± 2.1 bpm, and in the MIDA-90 sub-group it was 58.6 ± 7.5 bpm.

The mean arterial pressure during 50 or 90 min of ischaemia (anterior descending coronary artery occlusion) was 48.6 ± 10.9 mmHg in the SEVO-50 sub-group, 64 ± 8.5 mmHg in the MIDA-50 sub-group, and 47.7 ± 7 mmHg in the MIDA 90 sub-group.

The need for inotropes and events as asystole and ventricular fibrillation during ischaemia can be found in Table 2.

**Table 2:** Mortality, events, and inotrope administration during AMI induction.

	**Study A – 2020/2021**		
	**SEVO – 50**	**MIDA – 50**	**MIDA – 90**
Mortality	1/10	0/1	0/9
Asystole and/or ventricular fibrillation	6/9	1/1	4/9
Atropine administration	3/9	0/1	2/9
Adrenaline administration	3/9	1/1	0/9
Noradrenaline administration	1/9	1/1	0/9

Values are represented as number of animals/number of total animals. SEVO, sevoflurane; MIDA, midazolam.

### Cardiac function and infarct size

The CMR imaging results are summarized in Table 1. Animals from Study A sub-group SEVO-50 maintained their left ventricular ejection fractions (LVEF) within the normal range at both time points examined (54.7 ± 6.6% at 90 days and 51.4 ± 3.2% at 150 days post-procedure). Animals from Study A sub-group MIDA-90 had a reduced LVEF at both follow-up time points (39.1 ± 5.3% at 60 days and 38.5 ± 3.4% at 120 days post-procedure). The single animal in Study A sub-group MIDA-50 anaesthetized with midazolam and subjected to 50 min of ischaemia showed an LVEF of (47.2%) at 15 days post-procedure.

**Figure 1:**
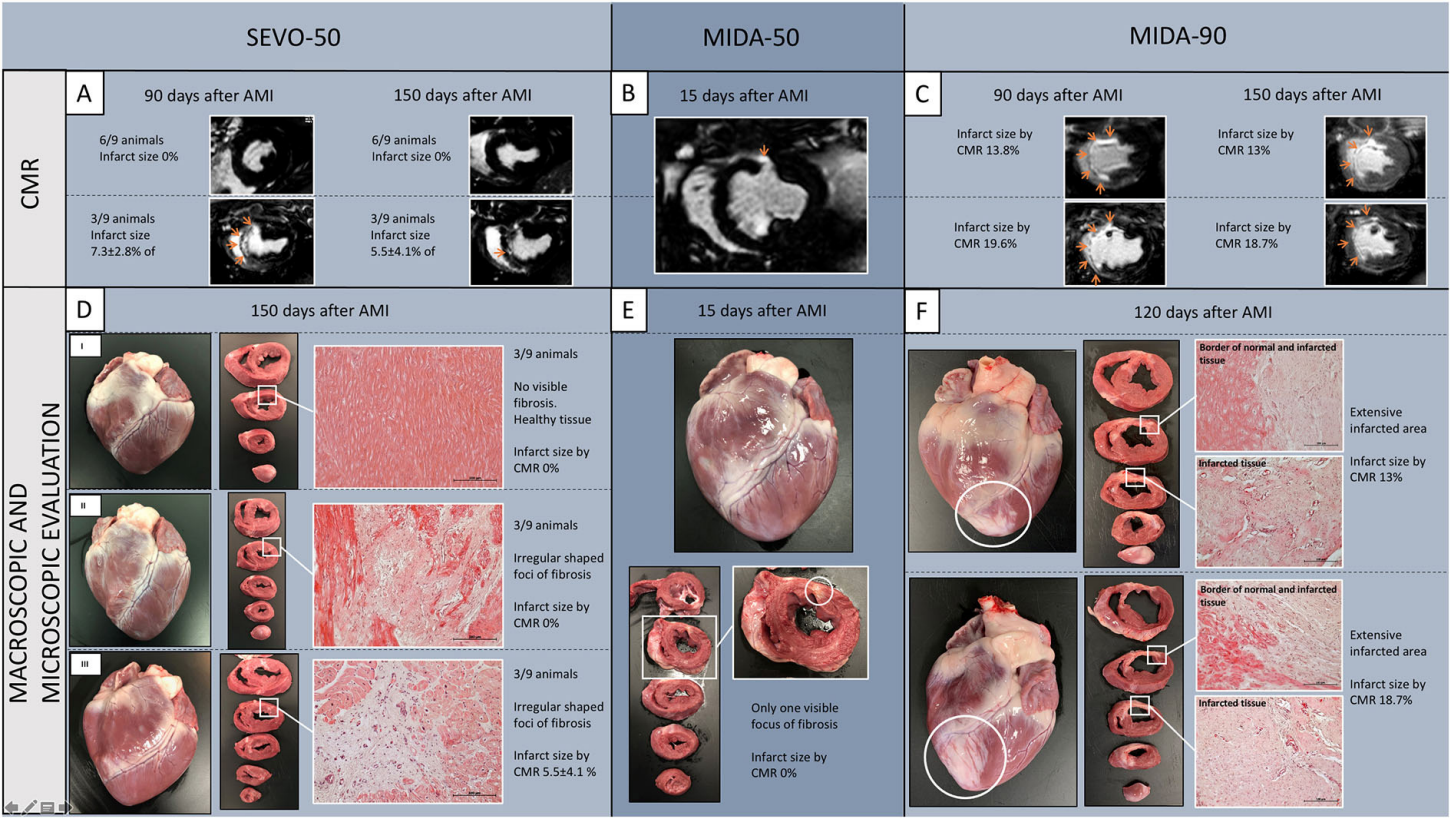
Late gadolinium-enhanced CMR representative images from Study A, sub-group SEVO-50 **(A)**, the single MIDA-50 animal **(B)**, and sub-group MIDA-90 **(C)**. CMR analysis showed infarcts were significantly smaller in sub-groups SEVO-50 and MIDA-50 compared to sub-group MIDA-90. Arrows depict late gadolinium-enhanced infarcted myocardium. **(D)** Macroscopic view of the anterior wall of the heart and transverse slices from apex to base showing an example of myocardial integrity devoid of any fibrotic areas (DI), an example of small areas of fibrosis (white box) with 0% of infarct size documented by CMR (DII), and an example of visible small areas of fibrosis with 5.1% (7.3 ± 2.8%, group SEVO-50, *n* = 3) of infarct size documented by CMR (DIII). **(E)** Small area of fibrosis visible in the fourth transverse heart slice in the single MIDA-50 animal. **(F)** Examples of extensive transmural infarction resulting in fibrosis. Representative histological sections were stained by haematoxylin and eosin (Original magnification 100x). SEVO, sevoflurane; MIDA, midazolam; AMI, acute myocardial infarction; CMR, cardiac magnetic resonance.

In agreement with the above LVEF results, animals from sub-group MIDA-90 had a significant area of LGE+ myocardium (infarcted) in the left ventricle (16.4 ± 4.6% at 60 days and 15.7 ± 3.2% at 120 days post-procedure). Conversely, animals in sub-group SEVO-50 that were maintained under anaesthesia with sevoflurane and had the LAD coronary artery occluded for 50 min displayed very limited areas of myocardial infarction (2.4 ± 3.9% at 90 days and 1.8 ± 3.4% at 150 days post-procedure). In the single animal in sub-group MIDA-50 subjected to 50-min LAD coronary artery occlusion but anaesthetized with midazolam, no infarcted tissue was detectable by CMR imaging at 15 days post-procedure. Figures 1A–C displays late gadolinium CMR images showing the differences in size of infarcts in each sub-group.

### Macroscopic and microscopic myocardial remodeling

Macroscopic evaluation of sub-group SEVO-50 hearts revealed, in six of nine animals, several small (~0.5–1.0 cm^2^) irregularly shaped fibrotic foci devoid of transmural extension into the interventricular septum and/or the anterior or lateral wall of the left ventricle. Three of the six animals with small areas of fibrosis did not show any LGE+ tissue in CMR imaging studies (Figure 1D). The three remaining hearts in this sub-group were devoid of any gross lesions. The single MIDA-50 animal had only one small focus of fibrosis in the anterior wall of the left ventricle (Figure 1E). All animals from sub-group MIDA-90 had variably sized foci of transmural myocardial infarction (pale fibrotic areas with thinning of the wall in some areas) involving areas of the septum, and anterior and lateral walls of the left ventricle from the apex to the base of the heart (Figure 1F).

Microscopic evaluation of all representative samples obtained from fibrotic foci showed deficits in the myocardium, presumably previously necrotic cardiomyocytes, replaced by fibrosis, and surrounded by very small numbers necrotic cardiomyocytes and inflammatory cells and variable amounts of neovascularization (Figure 1).

## Discussion

The novel data from our present study (Study A) along with our previous studies (B, C, and D) in a porcine model of induced AMI with respect to the induced myocardial infarction (as detected by CMR imaging, and macroscopic and microscopic evaluations) and subsequent LVEF show notable variations in the outcomes influenced by the anaesthetic protocol, duration of occlusion of LAD coronary artery, and breed of pig. For example, when subjecting a Landrace x Large White pig to 50-min LAD coronary artery occlusion using sevoflurane to maintain general anaesthesia, this resulted in minimal / non-detectable myocardial damage.

The cardioprotective potential of some anaesthetic agents, mainly gaseous, against ischaemic injury has been reported previously (5, 9, 13). However, the effectiveness of the protection of anaesthesia may be influenced depending on whether the protective agent is given before, during, or after induction of ischaemia or a combination of them. The present study addresses the effects of different anaesthetics agents used for maintaining general anaesthesia during the induction of myocardial ischaemia and the early phase of reperfusion. Several mechanisms have been proposed for the cardioprotective effects attributed to volatile anaesthetics such as the reduction in myocardial oxygen demand (in part attributed to reduced cardiac work) and better maintenance of energy stores or activation of pro-survival pathways, among others (13–16). However, the extent of this protective effect remains unclear. For this reason, the duration of ischaemia is critical to assess the potential cardioprotection by any anaesthetic agent. In a previous study from our group, 50-min LAD coronary artery occlusion/reperfusion resulted in a myocardial infarct and resultant fibrosis of ≈20% of the left ventricle at 60 days post-procedure (10) when using a combination of ketamine, midazolam, and xylazine (+ fentanyl) to maintain general anaesthesia. However, in Study A, the same occlusion protocol with sevoflurane resulted in minimal to no myocardial injury (2.4 ± 3.9% at 90 days) and normal left ventricle function. This protective effect is similar to that observed by Larsen et al. (17) in a similar porcine model of reperfused AMI (45-min occlusion of LAD coronary artery) that resulted in a 68% reduction in the infarct size when using sevoflurane to maintain general anaesthesia compared to pentobarbital (a non-cardioprotective anaesthetic agent). The 50-min occlusion of the LAD coronary artery in the single animal receiving midazolam, a benzodiazepine, to maintain general anaesthesia did not result in infarcted myocardium despite midazolam not being considered cardioprotective. However, activation of a benzodiazepine receptor protects the myocardium against ischaemia–reperfusion in rats (18), and this could partially explain our results. Another possible explanation for the lack of infarcted myocardium in the MIDA-50 mins occlusion animal is that the anaesthetic protocols used by our research group always combine the maintenance anaesthetic agent with a continuous infusion of an opioid (fentanyl) to alleviate intraprocedural pain which reduces the dose of anaesthesia required. Opioids can reduce myocardial injury associated with ischaemia and reperfusion, hypothetically *via* both cardiac and extra-cardiac activation of opioid receptors (19). However, this seems contrary to our previous results (10) of the same 50-min occlusion (+ reperfusion) procedure using midazolam in combination with ketamine and xylazine that included the same dose of fentanyl, yet resulted in clear irreversible myocardial damage. The different outcomes between both anaesthetic protocols could be explained by the dose of midazolam used as it was more than 10 times greater in study A. This could result in more efficient synergistic cardioprotective effects of midazolam and fentanyl in 50-min occlusion.

Another possible confounding factor is that the protocol using ketamine, midazolam, and xylazine (+ fentanyl) has only been used in Study B, which was performed in animals with a slightly different genetic background, pure Large White pigs instead of Large White x Landrace, and so a potential effect of breed cannot be discounted. In our experience, the most common farm swine breeds used for cardiovascular research in Europe include Landrace, Large White, Yorkshire, and Duroc, and their crosses. However, there are no studies directly comparing the effects of those genetic backgrounds on the outcomes of myocardial ischaemia.

We are aware that caution should be taken when comparing the combination of midazolam with a 50-min of occlusion of the LAD coronary artery and a different breed because there is only one animal in this group. The decision not to increase the number of animals in this protocol was motivated by the lack of induction of myocardial infarct, despite angiographically complete occlusion of the coronary artery, and the main goal of these pilot experiments was to establish a significant myocardial scar at 90 days post-induction that was to be treated with a novel regenerative therapeutic agent. Therefore, to avoid futile use of more animals, we decided not to continue with this protocol but studies of more animals would be required to confirm or refute the above-mentioned hypothesis.

The protocol that resulted in the desired infarct size was 90-min occlusion using midazolam (+ fentanyl) to maintain general anaesthesia. This was similar to our previous studies using 90 min of occlusion with propofol (+ fentanyl) (12), although a direct comparison of the infarct sizes is challenging due to the different methods used to determine them. The scar size at long-term follow-up was also similar to that obtained with ketamine, midazolam, and xylazine (+ fentanyl) with a 50-min occlusion in Large White pigs. Although xylazine (α-2 agonist) and midazolam (benzodiazepine) potentially have cardioprotective features, in this case, the combination of different anaesthetic agents allowed us to significantly reduce the dose of all the components, resulting in a hypothetically non-cardioprotective anaesthetic protocol.

This study has several limitations. First, we used juvenile pigs in all the studies due to logistic and methodologic reasons (manageable body size/weight of young animals) and budget limitations (high costs of adult minipigs). Myocardial infarction in humans is mainly a disease of the adult patient. Considerations should be taken when interpreting the results of AMI in young swine subjects as it might not completely mimic the pathology of adult subjects. Another limitation is that we only included female subjects in our study with the aim to be able to compare the outcomes with our previous works, with only female animals included. Current recommendations for animal research stress the need to use both female and male subjects in preclinical research for a more accurate translation to general human population. In the case of myocardial infarction, the effect of sex has been studied and it has been reported that the myocardium from females might be more protected against ischaemia, compared to males, but with worse myocardial remodeling (20, 21). Nevertheless, regarding preclinical research, a recent study shows that there are no differences in terms of infarct size and cardioprotection potential between female and male Göttingen minipigs (22). It was not designed to compare the effects of different anaesthetics on the outcome of induced AMI, and therefore, direct comparisons and statistical analyses are limited. Furthermore, we also discuss historical data of experiments performed by the same research group but in different research facilities, and therefore, changes in the equipment, housing, and general care of the animals may be confounding factors. Despite these limitations, we, as translational researchers, consider that these results are useful to avoid the use of animals in experiments that will fail to produce the AMI required for studies. The use of anaesthetic agents with faster recovery profiles, such as sevoflurane or desflurane, in preclinical research is encouraged to refine post-operative care and reduce anaesthesia-associated complications during weaning from mechanical ventilation because these are anaesthetic agents commonly used in clinical practice (23), which facilitates translation. However, based on our results and experience, and evidence from the literature, careful consideration should be taken when using the porcine model of reperfused AMI studies that have short durations of coronary occlusion and anaesthetic agents with potential cardioprotective features, such as sevoflurane. In addition, preclinical researchers should always be aware that each breed/lineage is endowed with specific features, namely cardiac sensibility/resistance to ischaemia.

## Data availability statement

The original contributions presented in the study are included in the article/supplementary material, further inquiries can be directed to the corresponding authors.

## Ethics statement

The animal study was reviewed and approved by Ethics Committee on Animal Experimentation of the Centre for Comparative Medicine and Bioimage (CMCiB) and by Generalitat de Catalunya (reference 10802).

## Author contributions

NS, JB, MA, FJ, CP, JR, MR, CG-A, XF, AG-A, MS, and MR performed the experimental works. NS, JB, and MR wrote the manuscript. BI, XF, MS, and MR provided funding. All authors reviewed and approved the published version of the manuscript.

## Funding

This research was funded by a grant (PI18/00277) from Instituto de Salud Carlos III (ISCIII), Spain—Fondo Europeo de Desarrollo Regional (FEDER). FJ is the recipient of the Ayudas para la formación de profesorado Universitario (FPU19/04925) grant from the Spanish Ministry of Science and Innovation. IDIBAPS belongs to the CERCA Programme and receives partial funding from the Generalitat de Catalunya.

## Conflict of interest

The authors declare that the research was conducted in the absence of any commercial or financial relationships that could be construed as a potential conflict of interest.

## Publisher's note

All claims expressed in this article are solely those of the authors and do not necessarily represent those of their affiliated organizations, or those of the publisher, the editors and the reviewers. Any product that may be evaluated in this article, or claim that may be made by its manufacturer, is not guaranteed or endorsed by the publisher.
